# Incidence and Prevalence of Cardiac Arrhythmias in Pericardial Syndromes

**DOI:** 10.31083/j.rcm2310347

**Published:** 2022-10-17

**Authors:** George Lazaros, Emilia Lazarou, Panagiotis Tsioufis, Stergios Soulaidopoulos, Aggeliki Valatsou, Maria Karmpalioti, Athanasios Sakalidis, Panayotis K. Vlachakis, Charalambos Vlachopoulos, Costas Tsioufis

**Affiliations:** ^1^First Cardiology Department, School of Medicine, Hippokration General Hospital, National and Kapodistrian University of Athens, 11527 Athens, Greece

**Keywords:** pericardial syndromes, arrhythmias, atrial fibrillation, myopericarditis, anticoagulation

## Abstract

Arrhythmias in pericardial syndromes have been poorly investigated and available data are mainly obtained from relevant studies however having different endpoints from arrhythmias. Thus, the incidence and prevalence of any type of arrhythmias may be actually higher than generally considered. Atrial arrhythmias, mainly atrial fibrillation and flutter have been reported as the most common rhythm disturbances in the setting of acute pericarditis. Concerning pathophysiology of atrial arrhythmias, in contrast to earlier hypothesis that they occur exclusively in the presence of an underlying structural heart disease, recent data support an arrhythmogenic potential of acute pericardial inflammation regardless of the presence of heart disease. In cases of myopericarditis, namely primarily pericarditis with evidence of myocardial involvement (i.e., troponin elevation without however overt left ventricular dysfunction and/or segmental wall motion abnormalities), ventricular arrhythmias appear to prevail. With reference to the rest of pericardial syndromes data on arrhythmias development are even more sparce. In particular, in constrictive pericarditis atrial tachyarrhythmias are the most commonly detected and seem to be related to disease severity and possibly to the underlying etiology. In this review we have summarized the available information on the incidence and prevalence of arrhythmias in pericardial syndromes. We wish to emphasize that the clinical significance of arrhythmias in this setting in terms of prognosis and optimal medical treatment (including need and safety of anticoagulation in atrial fibrillation/flutter complicating acute pericarditis), should be further investigated.

## 1. Introduction 

The most common pericardial syndromes include acute pericarditis (either in the form of a first attack or in the context of recurrent disease), constrictive pericarditis (permanent, transient or effusive-constrictive) and chronic pericardial effusion in the absence of overt inflammation (namely without C-reactive protein elevation) [1, 2, 3]. Myocardial involvement may complicate acute pericarditis at a rate that varies widely among studies, with a reported rate up to 25% [4, 5, 6]. In particular, the term “myopericarditis” is used in cases of primarily acute pericarditis with concomitant troponin elevation without however overt impairment of left ventricular contractility (ejection fraction) and/or segmental left ventricular wall motion abnormalities. In contrast, when the clinical features of myocarditis prevail, then the term of “perimyocarditis” is used [3].

Pericardial disorders, either isolated or in the form of myopericarditis, have attracted considerable attention in the era of SARS-CoV-2 pandemic, since they may appear either in the context of COVID-19 or after vaccination against SARS-CoV-2 especially with mRNA vaccine platforms [7].

Cardiac arrhythmias in the context of pericardial syndromes have been poorly investigated [1]. In this narrative review we have perused the available information in the international literature relevant to the presence, prognostic role and treatment of cardiac arrhythmias in patients with pericardial syndromes.

## 2. Isolated Acute Pericarditis 

As already mentioned, most data on the incidence of arrhythmic events during acute pericardial inflammation derive from studies not specifically designed for this purpose. The first report addressing the eventual link between acute pericarditis and arrhythmias dates back to 1956 [8]. In this investigation transient atrial fibrillation was reported in 4 out of 30 patients (13%) diagnosed with acute pericarditis, a rate which was exactly the same with that observed in a similar study conducted 4 years later in 31 patients with acute idiopathic pericarditis [9]. In 1962, in a clinicopathological study on hearts from 144 patients who succumbed from cardiac arrhythmias or conduction disturbances, acute pericarditis was detected in 38 cases (~26%) [10]. Notably, sinus node involvement was observed in all of the 38 cases, whereas atrial arrhythmias had been documented before death in 26 out of 38 cases (i.e., 68%) [10].

The first study that was specifically designed to investigate the association between acute pericarditis and arrhythmias was conducted in 1976 by D. Spodick [11]. In this study 100 consecutive patients with acute pericarditis of any etiology (idiopathic or secondary to specific causes such as uremic, neoplastic etc.) were enrolled. The protocol for arrhythmias detection included routine electrocardiograms (ECGs) with additional recordings in case of palpitations or other self-reported complaints, observations during regular physicians and nurse examinations (usually every 1 to 4 hours depending on the individual patient conditions) and continuous ECG monitoring in case of post myocardial infarction pericarditis. Notably, patients with cardiac tamponade were excluded from analysis. Arrhythmia was defined as 6 ectopic beats per minute or anything worse. According to this study results the overall incidence of clinically significant arrhythmia was 7% and arrhythmia in all cases was of supraventricular origin. In particular, 5 cases of atrial fibrillation have been recorded, 1 case of atrial flutter and 1 case of junctional tachycardia. The author emphasized that all arrhythmic events appeared in patients with an underlying heart disease involving myocardium, valves, or coronary arteries. Thus, this study showed that, arrhythmias in the setting of acute pericarditis are rare, exclusively supraventricular and are observed in patients with structural heart disease [11]. The author also emphasized that the underlying heart disorder was responsible for the development of heart rhythm disturbances rather than the propagation of inflammation from the visceral pericardium to the sinus node (which is actually located just beneath (1 mm) below the visceral pericardium at the right atrial region) [12]. This point of view has been further strengthened by a pathological study which failed to disclose extension of inflammation to the sinus node in a wide spectrum of acute pericarditis etiologies including pyogenic pericarditis, which is characterized by an intense inflammatory burden [13]. Nevertheless, in contrast to the latter data, as already mentioned sinus node involvement was observed in all cases (38 out of 38) of patients with acute pericarditis who died of arrhythmias or conduction system disturbances [10]. In the latter study it has also been shown that ganglia and nerve fibers in the region of sinus node and atrial free wall were involved in the inflammatory process. Taking into account all the above the arrhythmogenic potential of acute pericarditis remains controversial.

After this initial effort to address arrhythmias in pericarditis, the same author assessed the incidence of arrhythmias in patients with acute pericarditis with a more reliable methodology including continuous ambulatory ECG (Holter monitoring) in all cases [14]. The study population consisted of 50 patients with acute pericarditis of different etiologies. Among them, 49 were finally analyzed due to a non-reliable recording in 1 case. At enrollment all patients were in sinus rhythm. An underlying heart disease was present in 29 patients (most often acute myocardial infarction) and the remainder (20 patients) was not. Intermittent supraventricular arrhythmias were recorded in 4 patients (~8%) namely in 1 patient with heart disease and in 3 without. In addition, other non-sustained arrhythmias occurred in 8 out of 29 patients (28%) with heart disease and in no patient without structural heart disease. In line with his previous observations, the author once more suggested that arrhythmias in the setting of acute pericarditis imply a cardiac abnormality.

After several years of paucity of new data, in 2015 a large-sized study was published on the incidence, prognostic impact and treatment lines of new-onset atrial fibrillation/flutter in cases of acute pericarditis [15]. This study included 822 patients (mean age 53 ± 15 years, 444 men) enrolled in 2 referral centers for pericardial diseases in Italy and Greece (Maria Vittoria Hospital, Torino, and Hippokration General Hospital, University of Athens Medical School, respectively). Approximately 85% of patients were diagnosed with idiopathic/viral acute pericarditis. The high rate of idiopathic/viral forms reflects the shift in the epidemiology of pericarditis in recent years where a steep decrease in rheumatic, tuberculous (at least in the Western world) and post-acute myocardial infarction pericarditis (namely Dressler syndrome) has been observed, along with a substantial increase of post cardiac injury syndromes after cardiac surgery and implantation of cardiac electronic devices [1].

**Fig. 1.**
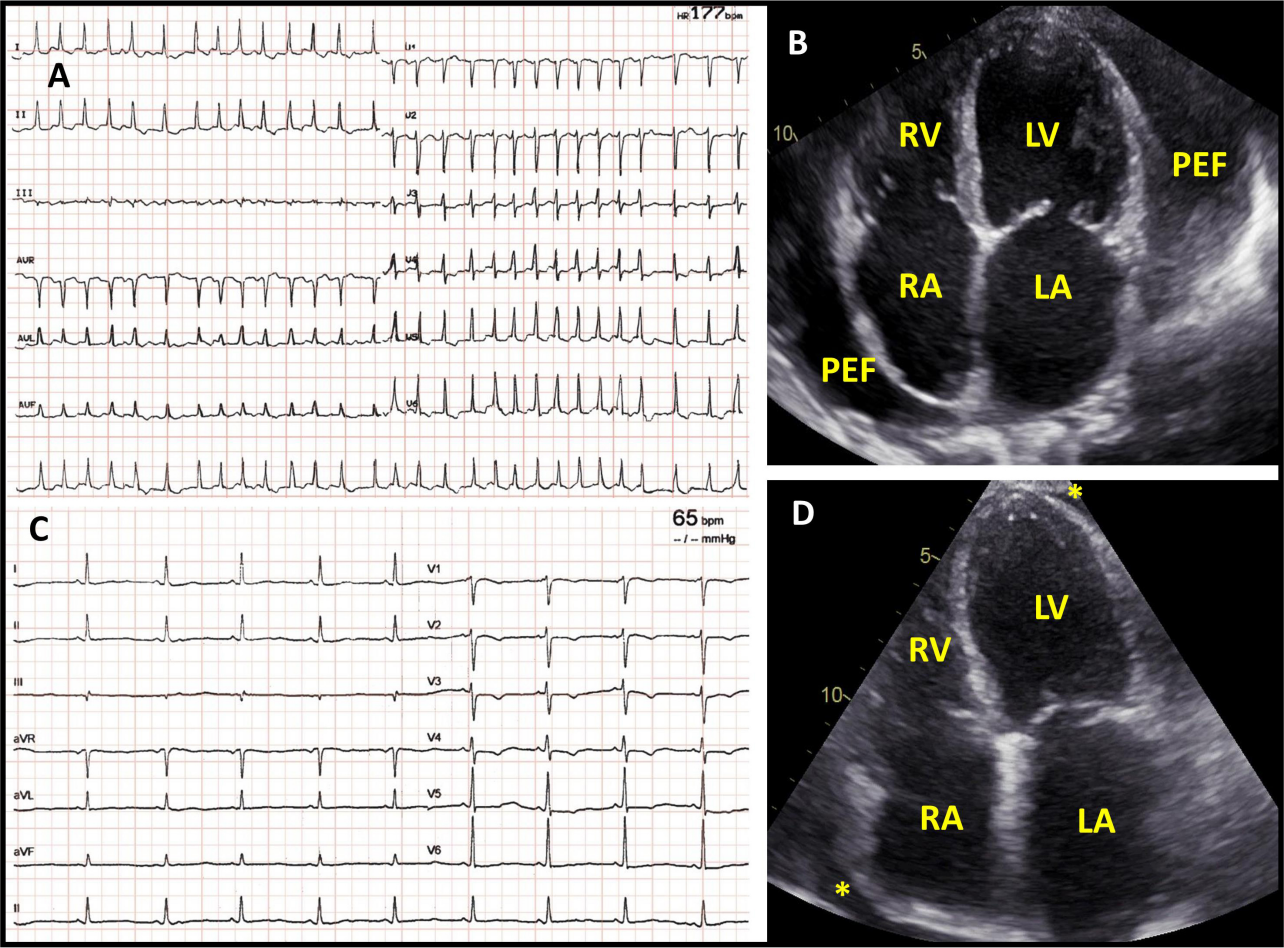
**Arrhythmias in patients with pericardial inflammation**. New-onset atrial fibrillation with rapid ventricular response (A), in a patient with acute pericarditis and large circumferential pericardial effusion (B), without signs of hemodynamic compromise. Arrhythmia converted spontaneously after 12 hours to sinus rhythm (C). Pericardial effusion regressed almost completely after 14 days of anti-inflammatory treatment (D). PEF, pericardial effusion; LV, left ventricle; RV, right ventricle; LA, left atrium; RA, right atrium; *Depicts residual pericardial effusion after anti-inflammatory treatment.

The diagnosis of acute pericarditis in the above-mentioned study was based on the contemporary European Society of Cardiology recommendations and the diagnosis of pericarditis-related atrial fibrillation/flutter was established in the presence of new-onset tachyarrhythmia (recorded by ECG or continuous ECG monitoring), that lasted ≥30 seconds. To minimize the possibility of silent (namely not recognized episodes of atrial fibrillation) all patients were strongly advised to report episodes of palpitations and in such cases Holter monitoring was scheduled if clinical assessment during the target episode did not reveal arrhythmias. According to this study results, atrial fibrillation or flutter were recorded in 4.3% of the overall population, namely in 35 patients (mean age 66.5 ± 11.3 years, 18 men) (Fig. 1). Patients with arrhythmia were older as compared to their counterparts without arrhythmia and presented more often with arterial hypertension, pericardial effusion and dilated left atrium on echocardiography. Notably, in contrast with the previously reported studies, structural heart disease was present in only 17% of patients who developed atrial fibrillation/flutter during the index attack of acute pericarditis.

Arrhythmia converted spontaneously to sinus rhythm in approximately ~75% of cases within 24 hours. Interestingly, during a 30-month follow-up, patients with arrhythmia in the acute phase depicted a significantly higher rate of atrial fibrillation/flutter recurrence during long-term follow-up compared to patients without arrhythmias (34.3% vs. 8.9%, *p *< 0.001). In the former patients, in 75% of cases arrhythmia recurred within 3 months after the index episode and in 75% on occasion of pericarditis recurrence.

Another worthy of mentioning finding in the latter investigation is that patients with arrhythmia in the acute phase did not depict a higher rate of complications in the long-run, namely constrictive pericarditis, cardiac tamponade, recurrent pericarditis, stroke/transient ischemic attack, any peripheral embolism and death. The decision of administering chronic oral anticoagulation to patients with atrial fibrillation/flutter was based on the contemporary guidelines on this arrhythmia [16]. Accordingly, anticoagulation was administered in approximately one third of patients based on the individual CHADs score, a practice that seems reasonable taking into account that in ~34% of the latter patients arrhythmia recured during follow-up. A possible concern relevant to the administration of anticoagulation during the acute phase of pericarditis, is the fear of intrapericardial hemorrhage and cardiac tamponade. In acute pericarditis the inflamed, hyperemic and rough pericardial layers may theoretically impose a high risk of hemorrhage eventually with catastrophic consequences in patients receiving anticoagulation. However, this possibility was not verified in this study since none of the anticoagulated patients developed clinically relevant intrapericardial hemorrhage. Another reassuring clue against any potential concerns about intrapericardial hemorrhage in patients with acute pericarditis is provided by an earlier study where 9 patients with acute myopericarditis misdiagnosed as acute myocardial infarction received thrombolytic therapy [17]. In these unfortunate cases, only 1 single patient developed a hemodynamically insignificant pericardial effusion which however regressed during follow-up. Putting together all the above, although the risk of developing pericardial hemorrhage under anticoagulation is at least theoretically present, the actual risk seems overestimated and in the presence of specific indications, anticoagulation should be administered based on the individual ischemic risk score.

In a more recent investigation where several clinical and laboratory findings were recorded in 175 patients hospitalized with a first episode of pericarditis, the reported rate of new-onset atrial/fibrillation/flutter in 175 patients was 8.2% [18]. In another pertinent study the incidence of new-onset atrial fibrillation/flutter was separately assessed according to age and sex. In this work which included 240 patients (141 males, aged 59.2 ± 18.4 years) the overall rate of the above-mentioned arrhythmias was 10% [19]. Although no significant sex-related differences were found in the incidence of arrhythmia, the observed rate of atrial fibrillation/flutter was higher in patients aged >60 years as compared to the younger counterparts (15.9% vs. 3.8%, *p* = 0.003). Notably, new onset atrial fibrillation/flutter has not been associated with impaired long-term prognosis in this and other works. Indeed, in two risk scores that have been published today for the prediction of recurrent or complicated pericarditis (defined as pericarditis with a non-idiopathic etiology, and/or requiring hospitalization and/or complications) atrial arrhythmias did not emerge as a variable associated with complicated course [20, 21]. In one of the latter risk scores which reported specifically on new-onset atrial fibrillation/flutter the recorded rate in the overall population studied (262 patients) was 9.5%, with the relevant rate being 13.3% in patients who depicted pericarditis recurrence vs 15.4% in those who did not (*p* = 0.768) [20].

Similar results with the previous study have been also reported in another contemporary investigation conducted in the United States of America, which included 240 patients with a first episode of pericarditis (idiopathic in 53%) [22]. The median follow-up was 179 days. The rates of cardiac tamponade, constrictive pericarditis, treatment failure, recurrent pericarditis and death during the study period were recorded. At least one of the adverse outcomes was recorded in 34% of patients. In the overall cohort the rate of atrial fibrillation was 26.3% (not specified whether new-onset or chronic). Among patients who experienced an adverse event the rate of atrial fibrillation was 24.4% whereas the relevant rate in patients with uncomplicated course was 27.2% (*p* = ns) [22].

Interestingly novel biomarkers have been recently reported to predict the development of atrial fibrillation/flutter in patients hospitalized with a first episode acute pericarditis. In particular, quantification of epicardial fat volume during computed tomography of the chest using a dedicated software has been shown to predict atrial fibrillation appearance during hospitalization. Indeed, higher epicardial fat volume was significantly associated with a higher rate of in-hospital atrial fibrillation. For a cut-off value of 123.5 ${\mathrm{cm}}^{3}$, epicardial fat volume had 83.3% sensitivity and 71.9% specificity for detecting patients who develop new-onset atrial fibrillation [23].

As already mentioned, SARS-CoV-2 infection is a new important chapter in medicine that has deeply influenced everyday life and social normalcy in the previous 3 years [24]. Remarkably inflammatory heart disease (such as pericarditis, myocarditis and mixed conditions such as myopericarditis and perimyocarditis) has been associated with adverse prognosis and may be caused either by SARS-CoV-2 infection or vaccination against the virus, especially with mRNA platforms [25]. The incidence of arrhythmias in the setting of SARS-CoV-2- or vaccine-induced inflammatory heart disease is difficult to estimate, since the concomitant severe respiratory infections, hypoxemia, sepsis, heart failure, renal failure, electrolytes imbalance etc. are potentially confounding factors. Actually, an increased risk of atrial fibrillation has been detected 15–21 days following a first dose of mRNA-1273 vaccine (IRR 2.06, 95% CI 1.11, 3.82) and ventricular fibrillation at 22–28 days following a second dose of ChAdOx1 vaccine (IRR 1.35, 95% CI 1.05, 1.74) [7]. Moreover, a higher rate of cardiac arrhythmias has been recorded after 1–7 days of a second dose of mRNA-1273 vaccine (IRR 2.32, 95% CI 1.49, 3.62). On the other hand, an increased risk of arrhythmias has also been observed in the 1–28 days following a SARS-CoV-2 positive test. Nevertheless, the specific rate of arrhythmias attributed to inflammatory heart disease cannot be estimated at present.

Last but not least, the presence of arrhythmias in patients with recurrent pericarditis is difficult to estimate since most patients are followed-up on an outpatient basis. As a result, eventual arrhythmic events may be missed. In a pertinent study assessing the long-term outcome in 61 difficult-to-treat patients with refractory recurrent pericarditis, atrial fibrillation was diagnosed in 5 patients (8.2%) during an average follow-up of 8.3 years [26]. Atrial fibrillation was transient in all instances and no patient developed chronic atrial fibrillation. This study highlights that a non-negligible subset of patients with recurrent pericarditis may develop transient episodes of atrial fibrillation during the course of the disease (especially during flares). Thus, these patients should be instructed to seek promptly medical advice in case of palpitations. This is important in terms of medical treatment since some of the above-mentioned patients may be candidates for chronic oral anticoagulation depending on the individual (ischemic) CHA2DS2-VASc score [15].

The most important studies addressing incidence and types of sustained arrhythmias in patients with isolated acute pericarditis are summarized in Table 1 (Ref. [8, 9, 10, 11, 14, 15, 18, 19, 20, 26]).

**Table 1.** **Most important studies addressing rates and types of sustained arrhythmias in patients with isolated acute pericarditis**.

		Publication year	Patients included	Atrial/supraventricular arrhythmias	Ventricular arrhythmias
Acute pericarditis					
	Scherl ND [8]	1956	30	13% atrial fibrillation	-
	Soffer A [9]	1960	31	13% atrial fibrillation	-
	James TN [10]	1962	38	68% atrial arrhythmias	-
	Spodick D [11]	1976	100	Supraventricular in 7% (6% atrial fibrillation/flutter)	-
	Spodick D [14]	1984	50	Supraventricular tachycardia in 8%	Paroxysmal ventricular tachycardia in 6%
	Imazio M *et al*. [15]	2015	822	Atrial fibrillation/flutter in 4.3%	-
	Lazaros G *et al*. [18]	2018	175	Atrial fibrillation/flutter in 8.2%	-
	Lazaros G *et al*. [19]	2021	240	Atrial fibrillation/flutter in 10%	-
	Lazarou E *et al*. [20]	2021	262	Atrial fibrillation/flutter in 9.5%	-
Recurrent pericarditis					
	Brucato A *et al*. [26]	2006	61	New onset atrial fibrillation in 8.2%	-

## 3. Arrhythmias in Myopericarditis 

Myocarditis and pericarditis are located at the edges of a wide spectrum of intermediate conditions [3]. Thus, myopericarditis encompasses cases that resemble isolated pericarditis in terms of clinical features, treatment recommendations and outcome. On the other hand, perimyocarditis shares similar features with “pure” myocarditis. Troponin elevation in cases of inflammatory heart disease who present with primarily pericarditic symptoms (myopericarditis) has been observed in ~14–32% of cases [4, 5, 27]. We wish to anticipate that according to the current evidence, troponin elevation in myopericarditis does not affect long-term prognosis and it should not be perceived as a negative prognostic marker in terms of complications as in acute coronary syndromes [1, 4, 5, 27]. Regarding treatment, the 2015 ESC guidelines recommend the lowest effective dose of non-steroidal anti-inflammatory medications for the shortest possible duration because previous studies depicted a more pronounced myocardial necrosis and adverse outcome in experimental animal studies with this treatment [1]. Moreover, in contrast to isolated pericarditis, clues favoring colchicine administration in myopericarditis are insufficient although recent data support its administration even in this context [1, 2, 3, 28]. Regarding recurrences, interestingly myopericarditis depicts a statistically significant lower rate of relapses as compared to isolated pericarditis (11 vs. 32%) [4].

The incidence of arrhythmias in cases of myopericarditis has not been extensively studied and only a few studies have specifically addressed this issue. Thus, since most available data are provided by works not specifically designed for arrhythmias detection, available data should be interpreted with caution.

The occurrence of arrhythmias and electrical conduction disorders in patients with myopericarditis and isolated pericarditis was assessed in a study which enrolled 50 patients (65% males, mean age 45.6 ± 15.7 years) [12]. All patients underwent endomyocardial biopsy which depicted isolated pericarditis in 40 cases (65% males, mean age 45.6 ± 15.7 years), and myocardial involvement (perimyocarditis or myocarditis) in 10 cases (70% males, mean age 46.1 ± 15.8 years) [12]. ECGs and 24-hour Holter recordings were used for arrhythmias detection. In the isolated pericarditis group atrial fibrillation was reported in 20% of cases, paroxysmal supraventricular tachycardia in 5%, ventricular tachycardia in 2.5%, whereas there were no cases of ventricular fibrillation. In the group with myocardial involvement, there were no cases of atrial fibrillation. Paroxysmal supraventricular tachycardia (other than atrial fibrillation) was found in 40% of cases, ventricular tachycardia in 10% and ventricular fibrillation in 20%. Notably, conduction disturbances occurred in 7.5% and 10% of pericarditis and myocarditis/myopericarditis cases respectively. The high rate of transitory atrial fibrillation reported in this study in the subgroup of patients with isolated acute pericarditis (namely 20%) in the absence of overt structural heart disease, questions the previously mentioned hypothesis suggesting that acute pericarditis is not arrhythmogenic and arrhythmias appear only in the presence of structural concomitant heart disease [11, 14, 15].

The similarities and differences between myopericarditis and viral pericarditis have been addressed in another larger study that included 274 patients. Among them, 234 were diagnosed with pericarditis and the remainder with myopericarditis [5]. Cardiac arrhythmias were observed in 16.7% and 65% of pericarditis vs myopericarditis cases respectively (*p *< 0.001). In specific, atrial fibrillation was detected in 2.5% of patients with myopericarditis and in 7.7% of those with isolated pericarditis (*p* = 0.380). The relevant rates of other supraventricular arrhythmias were 17.5% and 9% respectively (*p* = 0.175). On the other hand, ventricular arrhythmias (not further specified) were recorded in 40% and 0% in myopericarditis and pericarditis cases respectively (*p *< 0.001). Atrioventricular block developed in 5% of myopericarditis cases vs 0% in isolated pericarditis (*p* = 0.013). Left ventricular ejection fraction below 55% was found in 72.5% of patients with myopericarditis vs. 16% in acute pericarditis counterparts (*p *< 0.001). The presence of arrhythmias at baseline has been associated with the final diagnosis of myopericarditis (OR 17.6, 95% CI: 5.7, 54.1, *p *< 0.001).

Finally, in a large-sized investigation among 486 patients with inflammatory heat disease (median age 39 years, range 18–83 years, 300 men), 346 were diagnosed with pericarditis, 114 with myopericarditis and the remainder with perimyocarditis [4]. Supraventricular arrhythmias (type not specified) were recorded in 5.8% of isolated pericarditis cases and in 8.8% of myopericarditis (*p* = ns). Ventricular arrhythmias were observed in 0.3% and 4.4% of pericarditis and myopericarditis cases respectively (*p *< 0.001). A limitation of this work is that the adopted protocol for detecting cardiac arrhythmias has not been specified.

The above reported rates of sustained supraventricular and ventricular arrhythmias as well as conduction disturbances in patients with myopericarditis as compared to those with isolated acute pericarditis are summarized in Table 2 (Ref. [4, 5, 12]).

**Table 2.** **Comparison between rates of sustained supraventricular/ventricular arrhythmias and conduction disturbances in patients with myopericarditis compared to patients with isolated acute pericarditis**.

	Publication year	Patients with pericarditis/myopericarditis	Isolated pericarditis			Myopericarditis		
			SVA	VA	CD	SVA	VA	CD
Ristic *et al*. [12]	2000	40/10	25%	2.5%	7.5%	40%	30%	10%
Imazio *et al*. [5]	2008	234/40	16.7%	0%	0%	20%	40%	5%
Imazio *et al*. [4]	2013	346/114	5.8%	0.3%	NR	8.8%	4.4%	NR

SVA, supraventricular arrhythmias; VA, ventricular arrhythmias; CD, conduction disturbances; NR, not reported.

## 4. Cardiac Tamponade 

Cardiac tamponade is a life-threatening complication of pericarditis which if not promptly treated may lead to acute diastolic heart failure, cardiogenic shock and death. It complicates ~1.2% of acute idiopathic/viral pericarditis cases and approximately 20% of secondary pericarditis [29]. Actually, patients with cardiac tamponade have been in some instances excluded from studies focusing on the incidence of arrhythmias in acute pericarditis, in order to avoid confusion related to fluid-associated compression on the myocardium and coronary vessels [11]. Cases of atrial fibrillation/flutter during cardiac tamponade have been occasionally described in the literature [30, 31, 32]. Most important, a conversion of tachyarrhythmias to sinus rhythm during drainage of pericardial fluid has been described [33]. In clinical grounds, in patients with new-onset atrial fibrillation/flutter with rapid ventricular response and concomitant arterial hypotension, distant heart sounds, jugular veins distension as well as pulsus paradoxus, the possibility of cardiac tamponade should be considered before attributing hemodynamic instability exclusively to fast heart rate [33, 34].

## 5. Constrictive Pericarditis 

Constrictive pericarditis is characterized by thickening, fusion and finally calcification of pericardial layers due to long-standing pericardial inflammation [1, 35]. It complicates <1% of idiopathic/viral pericarditis and approximately 8% of specific pericarditis cases [29]. Constrictive pericarditis manifests clinically with symptoms and signs of peripheral stasis due to chronic diastolic heart failure (instead of acute which is the case in cardiac tamponade) [1, 2, 30]. Arrhythmias have been reported in patients with constrictive pericarditis and their prevalence depends mainly on the disease stage. In advanced stages, persistently elevated right atrial pressure may account for atrial arrhythmias [1]. Moreover, the underlying etiology of constrictive pericarditis may impact the prevalence and specific type of arrhythmias [29, 36, 37].

In a clinical investigation from the United States of America, 135 patients with surgically confirmed constrictive pericarditis were evaluated between 1985 and 1995 [36]. The most common underlying etiologies were idiopathic (~33%) followed by cardiac surgery, acute pericarditis and radiation (cumulative rate of the latter causes ~47%). The rate of atrial arrhythmias at baseline in this cohort of patients was 16% (22 patients) which is significantly lower as compared to historical controls from the same institution, namely 29% (*p *< 0.001). This difference was attributed to an earlier diagnosis of constrictive pericarditis in recent years along with a decrease in tuberculous forms. Atrial arrhythmias were the presenting symptom in 6 (4%) out of the 135 patients. Notably, in multivariate analysis atrial arrhythmias did not emerge as a predictor of outcome after pericardiectomy.

In another Italian retrospective study, among 500 consecutive patients with a first episode of acute pericarditis (~83 of idiopathic/viral etiology and 4% tuberculous), 9 finally developed pericardial constriction during a median follow-up of 72 months [29]. Among patients depicting pericardial constriction, atrial arrhythmia (not otherwise specified) was recorded in 2 patients (~22%).

Finally, in a retrospective study conducted in South Africa 121 patients requiring pericardiectomy due to definite or presumed tuberculosis in ~90% of cases, were enrolled [37]. At baseline atrial fibrillation was reported in 10 patients (8.3%). The cumulative perioperative mortality rate (mainly due to low cardiac output syndrome) was 14%. Atrial fibrillation at presentation was recorded in 6.7% of patients who survived after cardiac surgery vs 17.6% of those who died, with the difference being however not statistically significant (*p* = 0.13).

Interestingly, in a recent investigation including 91 patients who were hospitalized with the diagnosis of constrictive pericarditis at least 30 days after discharge from open heart surgery, history of atrial fibrillation emerged as an independent predictor of constrictive pericarditis development (*p* = 0.024) [38]. Finally, diastolic coronary artery compression by localized fibrous bands in constrictive pericarditis, has been anecdotally reported to cause arrhythmia due to myocardial ischemia [39].

## 6. Pericardial Effusion without Evidence of Inflammation

Chronic pericardial effusion without clinical and laboratory evidence of pericardial inflammation, namely without pleuritic chest pain and normal inflammatory markers such as C-reactive protein, is a common pericardial syndrome with a reported prevalence between 5.7 and 9% [40, 41]. Arrhythmias in this population have not been specifically addressed. Extinctic compression of myocardium and epicardial coronary arteries may in theory account for arrhythmias. However, this hypothesis has not been confirmed neither in the international literature nor in our institutional experience [42]. Thus, arrhythmias do not seem to be part of the clinical spectrum of patients with sterile pericardial effusion. Nevertheless, ambulatory ECG monitoring is not usually included in the diagnostic work-up protocol in this setting and thus, eventual arrhythmic events may not be detected. At present these patients should be investigated for arrhythmias on an individualized fashion based on patients’ history and reported symptoms (e.g., presence of palpitations, etc.).

## 7. Rare Pericardial Syndromes and Cancer-Related Pericarditis 

Arrhythmias and sudden death have been rarely described in rare pericardial syndromes such as large pericardial cysts and partial congenital pericardial defects which depict a frequency of ~1 per 100,000 and 0.01%–0.04% respectively [1, 43, 44]. In the specific context of pericardial cysts compression of adjacent structures has been encountered as the causative mechanism whereas herniation of ventricular myocardium and/or atrial appendages seems involved in small-sized pericardial defects [44, 45]. In particular, atrial arrhythmias, sometimes intractable, such as atrial fibrillation and flutter have been described in patients with pericardial cysts caused by atrial compression or impinging on a pulmonary vein or the sinus node region [46].

Another topic of increasing importance and interest in recent years is related to the development of pericardial syndromes in patients with cancer. In particular acute pericardial disease, pericardial effusion and myocarditis due to cancer per se or cancer therapies are included among the possible acute cardiovascular diseases encountered in those patients [1, 47]. As in other subgroups of pericarditis cases, acute pericardial syndromes may account for the onset of arrhythmias also in the subgroup of patients with cancer. However, it is often challenging to define the exact cause of arrhythmias in those patients since cancer treatment, myocardial involvement (myopericarditis) and eventual comorbidities (e.g., heart failure) may trigger arrhythmia onset [47]. In case that the cause-relationship between acute pericarditis/pericardial effusion and cancer treatments is highly suspected then treatment decisions should be taken through a multidisciplinary approach according to patient profile and clinical presentation.

## 8. Conclusions 

The rate of arrhythmias in patients with pericardial syndromes is non-negligible. Unfortunately, there are several unmet needs in this field that require additional research and clarification [48]. Current evidence supports the hypothesis that acute pericardial inflammation may trigger supraventricular arrhythmias (especially atrial fibrillation/flutter), both in the presence or absence of structural heart disease. Atrial fibrillation and flutter are most commonly encountered in patients with acute and constrictive pericarditis whereas ventricular arrhythmias are most often observed in cases with concomitant myocardial inflammation (myopericarditis). Unfortunately, rhythm disorders have not been systematically investigated in patients with pericardial syndromes and consequently the reported rate may not reflect their actual incidence and prevalence. Properly designed prospective studies are warranted to expand our knowledge on this topic and provide additional therapeutic tools for a tailored to the individual patient clinical management.
